# Keratin 14 is a novel interaction partner of keratinocyte differentiation regulator: receptor-interacting protein kinase 4

**DOI:** 10.3906/biy-1904-37

**Published:** 2019-08-05

**Authors:** Ceren SÜMER, Asiye Büşra BOZ ER, Tuba DİNÇER

**Affiliations:** 1 Department of Medical Biology, Institute of Health Science, Karadeniz Technical University, Trabzon Turkey; 2 Department of Medical Biology, Faculty of Medicine, Karadeniz Technical University, Trabzon Turkey

**Keywords:** Keratins, keratinocyte differentiation, RIPK4, protein-protein interactions

## Abstract

The epidermis, the outer layer of the skin, is formed by stratified keratinocyte layers. The self-renewal of the epidermis is provided by sustained proliferation and differentiation of the keratinocyte stem cells localized to the basal layer of the epidermis. Receptor-interacting protein kinase 4 (RIPK4) is an important regulator of keratinocyte differentiation, mutations of which are associated with congenital ectodermal malformations. In an attempt to identify the molecular basis of RIPK4’s function, we applied yeast two-hybrid screen (Y2H) and found basal layer-specific keratin filament component keratin 14 (KRT14) as a novel RIPK4-interacting partner. During keratinocyte differentiation, layer-specific keratin composition is tightly regulated. Likewise, the basal layer specific KRT14/keratin 5 (KRT5) heterodimers are replaced by keratin 1 (KRT1)/keratin 10 (KRT10) in suprabasal layers. The regulation of keratin turnover is under the control of signaling associated with posttranslational modifications in which phosphorylation plays a major role. In this study, we verified the KRT14-RIPK4 interaction, which was identified with Y2H, in mammalian cells and showed that the interaction was direct by using proteins expressed in bacteria. According to our results, the N-terminal kinase domain of RIPK4 is responsible for KRT14-RIPK4 interaction; however, the RIPK4 kinase activity is dispensable for the interaction. In accordance with their interaction, RIPK4 and KRT14 colocalize within the cells, particularly at keratin filaments associated with perinuclear ring-like structures. Moreover, RIPK4 did not show any effect on KRT14/KRT5 heterodimer formation. Our results suggest that RIPK4 may regulate the keratin turnover required for keratinocyte differentiation through interacting with KRT14.

## 1. Introduction

The epidermis is a stratified epithelium that protects the organism against environmental insults. The maintenance of the epidermis is provided by sustained proliferation and differentiation of the keratinocyte stem cells localized to the basal layer of the epidermis (Fuchs and Nowak, 2008; Blanpain and Fuchs, 2009). As keratinocyte stem cells commit to differentiate, they cease to proliferate and start to move upward to form suprabasal layers, including spinous, granular, and stratum corneum with different biochemical contents and morphologies (Fuchs and Nowak, 2008). In this differentiation process, transcription factor p63, which is the well-known master regulator of keratinocyte differentiation, regulates the expression of a plethora of proteins involved in keratinocyte differentiation and maintenance, including receptor-interacting protein kinase 4 (RIPK4), interferon regulatory factor 6 (IRF6), inhibitory kappa B kinase α (IKKα), and keratin 14 (KRT14) (Romano et al., 2007; Marinari et al., 2009; Thomason et al., 2010; Mitchell et al., 2012).RIPK4 is one of the major determinants of keratinocyte differentiation, mutations of which were previously associated with Bartsocas-Papas syndrome (BPS) that is characterized by severe congenital epidermal malformations (Kalay et al., 2012; Mitchell et al., 2012). In the epidermis of RIPK4-deficient mice, the disruption of keratinocyte differentiation leads to hyperplasia of the epidermis and misexpression of layer-specific keratinocyte markers (Holland et al., 2002; Rountree et al., 2010).Proteins rely on protein-protein interactions to perform their cellular function (Phizicky and Fields, 1995). The interaction of RIPK4 with transcription factor IRF6 and desmosomal protein plakophilin-1 (Pkp1) promotes keratinocyte differentiation through activation of keratinocyte-specific gene expression and regulation of desmosome signaling activity (Kwa et al., 2014; Lee et al., 2017). Therefore, in this study, we aimed to identify novel RIPK4-interacting proteins to clarify the molecular basis of RIPK4’s function in keratinocyte differentiation. For this purpose, a human keratinocyte cDNA library was screened by large-scale yeast two-hybrid (Y2H) assay and keratin filament protein KRT14 was identified as a RIPK4-interacting partner.Keratins, which are the main building blocks of the intermediate filaments of keratinocytes, are found in cells as obligate type I (KRT9-28) and type II (KRT1-8 and KRT71-74) heterodimers. These heterodimers align in an antiparallel fashion to form soluble tetramers that assemble into keratinocyte filaments (Fuchs and Weber, 1994; Kim et al., 2015). KRT14, which belongs to the type I keratin family, forms a heterodimer with type II keratin, KRT5. During keratinocyte differentiation, while the morphology and biochemical content of cells are changing, the keratin filaments’ composition is changing, as well. Likewise, KRT5/KRT14 heterodimers, which are expressed in the proliferative basal layer of the epidermis, are replaced by KRT1/KRT10 heterodimers in the spinous layer (Fuchs and Nowak, 2008). Basal layer-specific KRT5/KRT14 keratinocyte filaments are important for cell-cell (desmosomes) and cell-basal lamina (hemidesmosomes) attachments (Fuchs and Weber, 1994). Related to their function, KRT14 and KRT5 mutations are associated with epidermolysis bullosa simplex, an epidermal disorder characterized by fragility of basal keratinocytes and detachment of the cells from lamina upon physical trauma (Lane, 1994; Coulombe et al., 2009).As both KRT14 and RIPK4 are critical proteins important for keratinocyte differentiation and function, analysis of their interaction is critical for the understanding of the molecular basis of keratinocyte differentiation. Therefore, in this study we initially verified the RIPK4/KRT14 interaction, which we identified with Y2H screening, in mammalian and bacterial cells, and then further analyzed the interaction in terms of responsible RIPK4 domains and RIPK4 kinase activity. In order to gain insight into the biological significance of the interaction, we also investigated the effect of RIPK4 on KRT5/KRT14 heterodimer formation.

## 2. Materials and methods

### 2.1. Expression vectors and cloning

For the Y2H screen, the N-terminal kinase domain of RIPK4 (1-340) was subcloned from pCR3 VSV-N-RIPK4 (Meylan et al., 2002) into Gal4-DNA-BD containing pGBKT7 (Clontech, USA) yeast expression plasmid by using EcoRI-XhoI and EcoRI-SalI (Invitrogen, USA) restriction enzymes, respectively. For the construction of the mammalian and bacterial expression vectors, the gateway cloning system technology (Thermo Fischer Scientific, USA) was applied. Open reading frames (ORFs) of human full-length (FL) RIPK4 (NM_020639.2) and kinase-dead RIPK4 (K51R) were amplified from pCR3 VSV-RIPK4 (Meylan et al., 2002) by PCR with primers suitable for gateway cloning. Amplified RIPK4 and K51R ORFs were cloned into the p3xFlag CMV/DEST expression vector (Invitrogen, USA) to provide expression of RIPK4 and K51R in mammalian cells in fusion with the N-terminal Flag tag. Additionally, RIPK4 was cloned into pDEST-27 (Invitrogen, USA) and pcDNA-DEST53 (Invitrogen, USA) expression vectors for the expression of RIPK4 in mammalian cells in fusion with N-terminal GST and GFP tags, respectively. ORFs belonging to RIPK4 domains—RIPK4 N-Ter (containing kinase domain, aa 1-292), ID (aa 278-438), and C-Ter (containing ID and ankyrin, aa 278-784)—were amplified from the p3xFlag-RIPK4 expression vector and cloned into the p3xFlag CMV/DEST expression vector (Invitrogen, USA). Moreover, RIPK4 was cloned into the pDEST17 bacterial expression vector (Invitrogen, USA) to be expressed in bacteria in fusion with the N-terminal His tag. ORFs of human KRT14 (NM_000526.5) and KRT5 (NM_000424.4) were amplified by using the cDNA obtained from the human keratinocyte HaCaT cells. KRT14 was cloned into the pDEST-27 mammalian expression vector (Invitrogen, USA) and pGEX-KG/DEST bacterial expression vector (Invitrogen, USA) in fusion with the N-terminal GST tag. KRT5 was cloned into p3xFlag CMV/DEST (Invitrogen, USA) in fusion with the N-terminal Flag tag. All clones were verified with Sanger sequencing using a genetic analyzer (Applied Biosystems, USA).

### 2.2. Yeast two-hybrid screen

As bait, the N-terminal region of RIPK4 was cloned into the pGBKT7 yeast vector (Clontech, USA) in fusion with the Gal4 DNA binding domain, and the construct was sent to the German Cancer Research Center (DKFZ, Genomics and Proteomics Core Facilities - Protein Interaction Screening W150, Germany) for a large-scale Y2H screen. The human keratinocyte cDNA library, which was expressed in fusion with the Gal4 activation domain from vector pACT2.2 (Clontech, USA), was used as prey. Bait and prey proteins were expressed together in the AH109 yeast strain (Clontech, USA) and positive clones were identified by activation of HIS3 and MEL1 reporters that are controlled by GAL promoters. The Y2H screen was repeated three times with 0.4 mM, 0.2 mM, and 0.1 mM 3-aminotriazole (3AT) containing selective medium to determine strong and weak interactions. Library inserts were isolated by PCR and analyzed by DNA sequencing.

### 2.3. Cell lines, cell culturing, and transfections

HEK293T (human embryonic kidney cells), HaCaT (immortalized human keratinocytes), and HeLa (human cervix adenocarcinoma cell line) cells were cultured in Dulbecco’s modified Eagle’s medium supplemented with 10% heat-inactivated FBS and penicillin/streptomycin (100 µg/mL of each at 37 °C in a humidified atmosphere of 5% CO2). For a transient transfection of HEK293T, HeLa cells, and HaCaT cells, a standard calcium phosphate protocol (Jordan et al., 1996), DreamFect Gold Transfection Reagent (Ozbiosciences, USA), and Lipofectamine 2000 reagent (Thermo Fischer Scientific, USA) were used, respectively. 

### 2.4. Application of CRISPR/Cas9 system for knockout of RIPK4

The CRISPR/Cas9 system was applied to generate the mutant HaCaT cell lines lacking RIPK4 expression as described (Ran et al., 2013). RIPK4-specific guide RNA (g-RNA), which precedes the Protospacer Adjacent Motif (PAM), 5’ NGG, was designed using Benchling and CHOP-CHOP Web software. The applied g-RNA (5’-TCGACGCGGGCGAGTTCAC-3’) targets the region in exon 1 (between the 50th and 68th nucleotides) that corresponds to the beginning of the RIPK4 kinase domain (between the 66th and 849th nucleotides) (Supplementary Figure  1A). RIPK4-specific g-RNA was cloned into the Cas9-expressing plasmid, pSpCas9(BB)-2A-Puro (PX459) V2.0 (Addgene #62988, USA) and transfected into the HaCaT cells by Lipofectamine 2000 reagent (Thermo Fischer Scientific, USA). After 3 days of selection with puromycin (1 µg/mL), clonal cell lines were obtained by expanding the single cells in a regular maintenance medium. To determine the extent of CRISPR/Cas9-introduced mutations in the *RIPK4* gene, PCR amplified exon 1 was analyzed by sequencing. RIPK4 expression levels were analyzed in mutant HaCaT clones with western blot using anti-RIPK4. The clone named as RIPK4 KO was used in this study, considering the higher efficiency of RIPK4 depletion (Supplementary Figure  1B).

**Figure 1:**
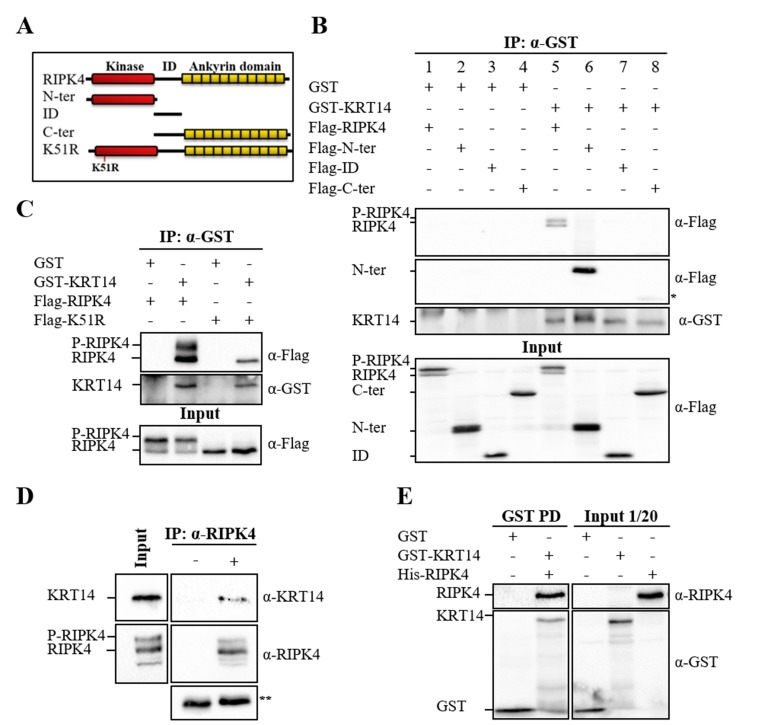
Interaction of RIPK4 with KRT14. RIPK4 constructs, used in interaction assays, were schematized with domains (A). HEK293T cells were transfected with indicated constructs. KRT14 was immunoprecipitated using anti-GST antibody followed by western blotting with anti-Flag and anti-GST antibodies (B, C). RIPK4 was immunoprecipitated using anti-RIPK4 in HaCaT cells. Rabbit anti-Flag antibody was used as a control. The lysate was analyzed by western blotting using anti-KRT14 and anti-RIPK4 antibodies (D). His-RIPK4 containing lysate was incubated with GST-KRT14 bounded beads and interaction was analyzed by western blotting using anti-RIPK4 and anti-GST (E). Input indicates total lysate. α represents anti. * represents nonspecific band. ** represents heavy chains of Flag (rabbit) and RIPK4 antibody, respectively.

### 2.5. Antibodies

The following antibodies were used: anti-KRT14 (Abcam, UK; Cat. No. ab7800), anti-KRT5 (Abcam, UK; Cat. No. ab52635), anti-RIPK4 (Santa Cruz Biotechnology, USA; Cat. No. sc-83320), anti-GST (Santa Cruz Biotechnology, USA; Cat. No. sc-459), anti-Flag M2 (Sigma-Aldrich, USA; Cat. No. F3165), secondary antibody HRP-conjugated antimouse (Bio-Rad, USA; Cat. No. 170-5047), antirabbit (Bio-Rad, USA; Cat. No. 170-5045), secondary antimouse-Cy3 (Abcam, UK; Cat. No. ab97035), and secondary antirabbit-Alexa Fluor 488 (Abcam, UK; Cat. No. ab150061).

### 2.6. Cell lysis and western blotting 

Cells were washed with ice-cold PBS and then lysed using TNTE buffer (50 mM Tris-Cl [pH 7.4], 150 mM NaCl, 1 mM EDTA, 1% Triton X-100, 10% glycerol, 5 mM Na4P2O7, 2 mM Na3VO4, 20 mM NaF, 1 mM PMSF, and 1X protease inhibitor cocktail tablet [Roche, Switzerland]) on ice for 30 min. The lysates were centrifuged at 16,000 × *g* for 15 min and protein concentrations were measured using a BCA protein assay kit (Thermo Fischer Scientific, USA). Beads were boiled in SDS-PAGE loading buffer (0.25 M Tris-CI [pH 6.8], 10% SDS, 50% glycerol, 0.01% Bromophenol Blue [Merck, Germany]) and then loaded on 7.5% SDS-PAGE gels. After proteins were transferred to a nitrocellulose membrane (Bio-Rad, USA), the membrane was blocked with 5% BSA in Tris-buffered saline (TBS) with 0.05% Tween 20. Indicated primary and secondary antibodies were used according to the manufacturer’s instructions. Protein bands were visualized with the ChemiDoc MP Imaging System (Bio-Rad, USA).

### 2.7. Coimmunoprecipitation 

The cell lysate was incubated with 1 µg of anti-GST and anti-Flag antibody for 2 h at 4 °C to immunoprecipitate (IP) GST-KRT14 and Flag-KRT5 proteins. Then 20 µL of equilibrated protein G agarose beads (Invitrogen, USA) were added to the samples and tubes were further incubated for 2 h at 4 °C with constant rotation. For immunoprecipitation of endogenous RIPK4, 1.7 mg of total protein containing cell lysate was incubated with 3 µg of anti-RIPK4 for 2 h at 4 °C. Then 30 µL of equilibrated protein G agarose, which was previously blocked with 0.1% BSA, was added to the samples and the samples were further incubated for 2 h at 4 °C with constant rotation. After the beads were washed five times with a wash buffer (50 mM Tris-Cl [pH 7.4], 150 mM NaCl, 1 mM EDTA [pH 8.0], 0.1% Triton X-100), the proteins were eluted with SDS-PAGE loading buffer and analyzed by western blot.

### 2.8. Bacterial expression of GST/His proteins and pull-down assay

His-RIPK4 and GST-KRT14 were transformed into *E. coli* BL21 DE3 and BL21 AI strains, respectively, and grown at 37 °C until the OD600 of the culture reached 0.6. The bacterial cells were treated with 0.2% L-arabinose and 100 mM IPTG (isopropyl 1-thio-D-galactopyranoside) for 2 h and 4 h at 20 °C to induce expression of His-RIPK4 and GST-KRT14, respectively. GST-, GST-KRT14-, and His-RIPK4-expressing bacterial cells were lysed in ice-cold lysis buffer (10 mM Tris-Cl [pH 7.5], 150 mM NaCl, 0.5 mM EDTA [pH 7.0], 0.5% Nonidet P-40, 1 mM PMSF). GST and GST-KRT14 were bound to GST trap beads (Chromotek, Germany) and the samples were incubated with His-RIPK4 containing bacterial cell lysate for 1 h at 4 °C. After the beads were washed with a wash buffer (10 mM Tris-Cl [pH 7.5], 300 mM NaCl, 0.5 mM EDTA, and 0.1% NP-40) five times at 4 °C, the proteins were eluted from the beads by using 0.2 M glycine-HCl (pH 2.5) and analyzed by western blot.

### 2.9. Fluorescence microscopy

The FLAG-RIPK4- and GST-KRT14-expressing vectors were transfected into the HeLa and HaCaT cells. At 24 h after transfection, the cells were fixed with 4% paraformaldehyde in phosphate-buffered saline (PBS) for 10 min and permeabilized with 0.2% Triton X-100 in PBS for 5 min. Between each step, cells were washed five times with PBS. The samples were blocked in 10% goat serum in PBS before addition of primary antibodies, anti-FLAG (1:1250), and/or anti-GST (1:500), which were diluted in 10% goat serum. After 90 min of incubation at room temperature (RT) with primary antibodies, the samples were washed five times with PBS and then incubated with secondary antibodies for 90 min at RT. Antimouse-Cy3 (1:1500) and antirabbit Alexa Flour 488 (1:1000), which were diluted in 10% goat serum, were used as secondary antibodies. After washing with PBS five times, the samples were mounted with ProLong Gold Antifade reagent containing DAPI (Thermo Fischer Scientific, USA). Images were acquired using a fluorescent microscope (Nikon, Japan).

### 2.10. Statistical analysis

Statistical significance values of the expression data were determined by Student’s t-test. P < 0.001 was considered significant.

## 3. Results

### 3.1. RIPK4 interacts with keratin 14 

RIPK4, which encodes a 784-amino-acid protein with a molecular weight of 87 kDa, has a conserved N-terminal serine/threonine kinase domain, an intermediate domain, and C-terminal ankyrin repeat domains (Figure  1A) (Meylan, 2002). In this study, we applied a large-scale Y2H screen in order to identify possible RIPK4-interacting proteins. During the Y2H screen, a human keratinocyte cDNA library was screened using the N-terminal RIPK4 kinase domain as bait. As a result of the screen, KRT14 was identified as a strong candidate that shows interaction even at highly stringent conditions (0.4 mM 3-AT) (data not shown). Since the Y2H screen was performed with the N-terminal region of RIPK4 and the cDNA clone gathered from the screen was corresponding to the partial ORF of KRT14, we analyzed whether full-length KRT14 and RIPK4 interact in mammalian cells as well. For this purpose, HEK293T cells were transfected with full-length Flag-tagged RIPK4 and GST-tagged KRT14 either alone or together, and the interaction was determined by coimmunoprecipitation (Co-IP) assay. The data presented in Figure  1B (lane 5) showed that the full-length RIPK4 and KRT14 specifically interact in mammalian cells as well. In this particular experiment, we also analyzed the RIPK4 domain(s) responsible for the KRT14 interaction (Figures  1A and  1B). According to our results, the kinase domain containing the N-terminal region of RIPK4 (N-ter) specifically interacted with KRT14 (Figure  1B, lane 6). On the other hand, the intermediate domain of RIPK4 (ID) (Figure  1B, lane 7) and intermediate and ankyrin domain-containing C-terminal region of RIPK4 (C-ter) (Figure  1B, lane 8) showed no interaction with KRT14. In order to test whether the kinase activity of RIPK4 is required for KRT14 interaction, the Co-IP experiment was repeated with kinase-dead K51R. According to our results, K51R continued to interact with KRT14 (Figure  1C, lane 4). As indicated in Figure  1B (lanes 5 and 6), kinase domain-intact full-length RIPK4 and N-terminal RIPK4 were detected as double bands, phosphorylated (P-RIPK4) and unphosphorylated (RIPK4) forms, because of RIPK4’s autophosphorylation activity (Bähr et al., 2000). In accordance, kinase-dead K51R appeared as a single band (Figure  1C, lane 4). Since the detected interaction between RIPK4 and KRT14 may be due to nonphysiological overexpression levels, the interaction was also confirmed for endogenous RIPK4 and KRT14 with Co-IP by using protein-specific antibodies. The result of endogenous Co-IP indicated that RIPK4 interacts with KRT14 in keratinocytes under physiological conditions as well (Figure  1D). Next, we intended to explore whether the interaction between RIPK4 and KRT14 is direct or part of a complex. For this, GST-KRT14, which was produced in bacteria, was immobilized to the beads and a GST pull-down assay was performed by using His-RIPK4-expressing bacterial lysate. GST alone was used to assess nonspecific binding as a control. The results showed that RIPK4 interacts with KRT14 directly (Figure  1E). 

### 3.2. RIPK4 colocalizes with KRT14

**Figure 2:**
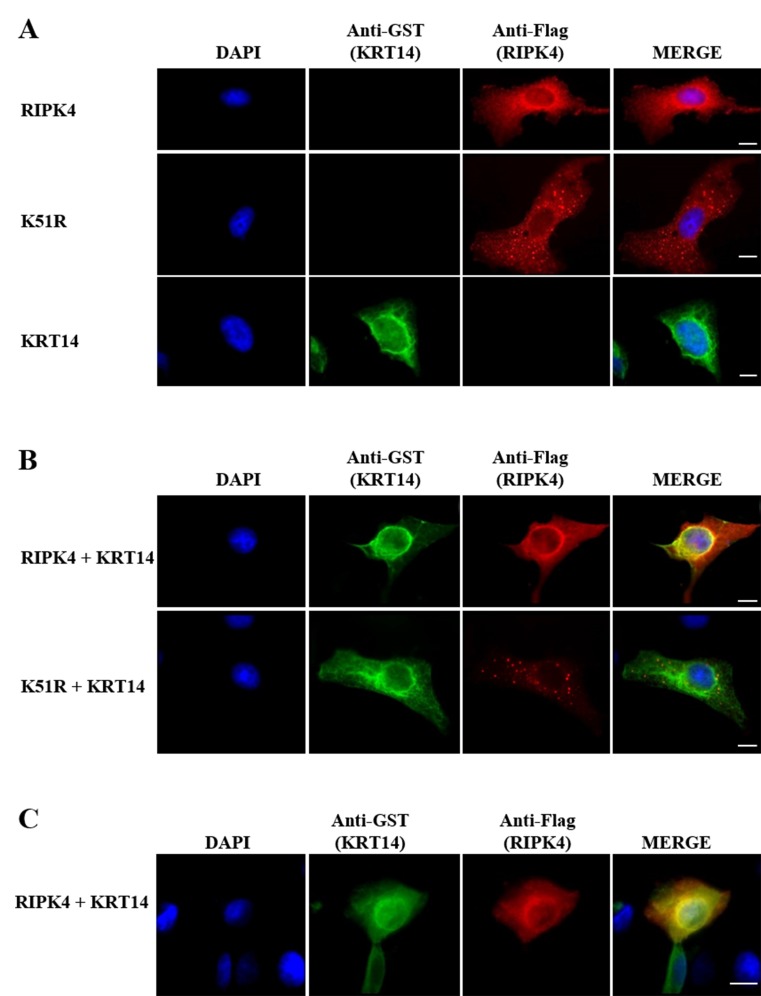
Colocalization of RIPK4 with KRT14. HeLa cells were transfected with Flag-RIPK4, Flag-K51R, or GST-KRT14 alone (A) or together (B). HaCaT cells were transfected with Flag-RIPK4 and GST-KRT14 together (C). Cells were stained with anti-KRT14 (green) and anti-Flag (red) antibodies.

In order to analyze the subcellular localization of RIPK4 and KRT14, an immunofluorescence assay was performed in HeLa cells. In this assay, the cells expressing Flag-RIPK4 and GST-KRT14 either alone (Figure  2A) or together (Figure  2B) were fixed and analyzed. K51R was also included in the experiment to analyze the effect of kinase activity on the subcellular localization of RIPK4 and KRT14. As shown in Figure  2A, when expressed alone, KRT14 showed typical keratin filaments staining, which shows a cytoplasmic reticular pattern concentrating around the periphery of the nucleus. On the other hand, RIPK4 appeared as tiny dot-like structures diffused in the cytoplasm, mainly around the nucleus. Different from RIPK4, K51R showed coarse dot-like structures scattered through the cytoplasm without surrounding the nucleus (Figure  2A). When expressed together, RIPK4 showed colocalization with KRT14, particularly at the ring-like structure around the periphery of the nucleus (Figure  2B). On the other hand, K51R did not colocalize with KRT14 (Figure  2B).RIPK4 and KRT14 showed the same colocalization pattern in keratinocyte HaCaT cells as well (Figure  2C). 

### 3.3. RIPK4 does not interact with KRT5 and has no effect on KRT5/KRT14 interaction

**Figure 3:**
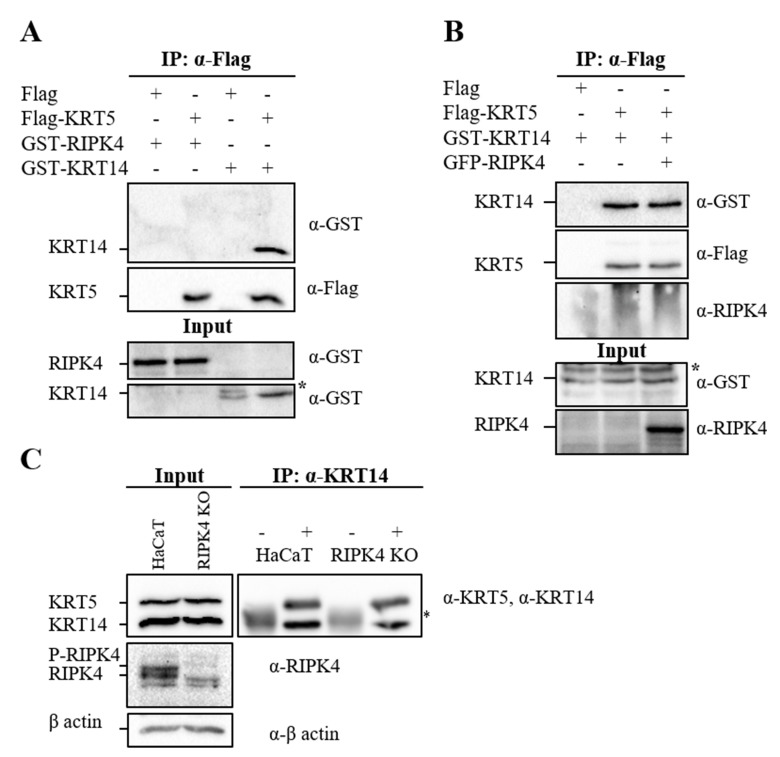
Effect of RIPK4 on KRT14/5 heterodimer formation. KRT5 was immunoprecipitated using anti-Flag antibody followed by western blotting with anti-Flag and anti-GST antibodies (A). KRT5 was immunoprecipitated using anti-Flag antibody from the cell lysate of KRT14 alone and KRT14/RIPK4 together with overexpressed HEK293T cells. Western blot was performed with anti-Flag, anti-GST, and anti-RIPK4 antibodies (B). KRT14 was immunoprecipitated from HaCaT and RIPK4 KO cell line using anti-KRT14 antibody (+). Mouse anti-Flag antibody was used as a control (-). Western blot was performed with anti-KRT5, anti-KRT14, and anti-RIPK4 antibodies (C). Input indicates total cell lysate. α represents anti. * represents nonspecific bands.

KRT14 and KRT5 form parallel coiled-coil heterodimers to build a dynamic cytoskeleton in the basal layer of keratinocytes (Fuchs and Weber, 1994). As we showed the interaction between RIPK4 and KRT14, we also analyzed whether RIPK4 interacts with KRT5 with Co-IP assay. In this experiment, KRT5 was immunoprecipitated and the presence of KRT14 and RIPK4 in the complex was analyzed. As expected, KRT5 interacted with KRT14; however, there was no interaction between KRT5 and RIPK4 (Figure  3A, IP, panel 1, lanes 2 and 4). Then we analyzed the effect of RIPK4 overexpression and depletion on KRT5/KRT14 interaction by Co-IP assay (Figures 3B and 3C). The RIPK4 overexpression, which was detected by anti-RIPK4 in total lysates (Figure  3B, input, panel 2, lane 3), did not show any effect on KRT5 and KRT14 interaction (Figure  3B, IP, panel 1, lanes 2 and 3). Similar to the result shown in Figure  3A, RIPK4 did not show any interaction with KRT5 (Figure  3B, IP, panel 3, lane 3). The CRISPR/Cas9 strategy was applied to deplete RIPK4 expression in HaCaT cells. The mutant HaCaT cell line (RIPK4 KO), in which RIPK4 expression is significantly suppressed (Supplementary Figure  1B), carries heterozygous RIPK4 mutations. In one allele, one nucleotide insertion (66th base) resulted in a premature stop codon that led to knockout, while the other allele contained an in-frame deletion of 12 nucleotides (between the 60th and 73rd nucleotides) (data not shown). Even though the 12-nucleotide deletion encoded a protein, our immunoblot analysis showed that RIPK4 KO had approximately 20% of RIPK4 protein compared to the parental cell line, which indicates that deletion destabilizes the protein (Supplementary Figure  1B).Therefore, in order to analyze the effect of RIPK4 depletion on KRT5/14 interaction, endogenous KRT14 was immunoprecipitated from the RIPK4 KO cell line by using anti-KRT14 and its interaction with KRT5 was analyzed by western blot using anti-KRT5. As shown, in total lysates, RIPK4 expression was efficiently suppressed in RIPK4 KO cells (Figure  3C, input, panel 2, lane 2) and depletion of RIPK4 did not affect the interaction between KRT5 and KRT14 (Figure  3C, IP panel, lane 4).

## 4. Discussion

The epidermis provides an essential barrier against the external environment and its continuity is maintained with epidermal homeostasis, which is a delicate balance between proliferation and differentiation of keratinocytes (Fuchs and Nowak, 2008). RIPK4 is one of the key proteins involved in keratinocyte differentiation (Holland et al., 2002; Rountree et al., 2010). Consequently, its deficiency in humans and mice leads to severe epidermal developmental defects (Holland et al., 2002; Kalay et al., 2012). Despite its critical role in keratinocyte differentiation, little is known about how RIPK4 performs its function in this process. Identification of protein interacting partners gives important clues about the functions of proteins (Phizicky and Fields, 1995). As the Y2H system offers us an approach for the identification of the interaction partners of proteins (Fields and Song, 1989), we applied the Y2H screen and identified KRT14 as an interaction partner of RIPK4. KRT14 is a member of intermediate filaments restricted to basal-layer keratinocytes (Fuchs and Weber, 1994), and similar to RIPK4, its expression is induced by the keratinocyte fate-determinant factor, p63 (Romano et al., 2007). KRT14 is connected to desmosomes through interacting with desmosomal structural protein PKP1 (Hofmann et al., 2000). Interestingly, Pkp1 has been recently shown to be involved in keratinocyte differentiation through its interaction and phosphorylation by RIPK4 (Lee et al., 2017). All of this information points to the biological importance of the RIPK4 and KRT14 interaction in keratinocyte differentiation. Therefore, in this study, the RIPK4/KRT14 interaction, which was identified by Y2H screen, was confirmed in mammalian and bacterial cells (Figures 1B and 1D) and was demonstrated to be direct (Figure  1E). Ankyrin repeats, which are located at the C-terminus of RIPK4, are well known for their roles in protein-protein interactions (Sedgwick and Smerdon, 1999). However, the interaction between RIPK4 and KRT14 was particularly mediated by RIPK4’s N-terminal kinase domain (Figure  1B). Previously, RIPK4’s kinase activity was shown to be dispensable for RIPK4 interaction with IRF6 (Huang et al., 2018). Similar to this report, our result showed that RIPK4 kinase activity is not necessary for RIPK4/KRT14 interaction (Figure  1C). Proteins localize to the same cellular compartments to perform their functions (Loo et al., 2014). RIPK4 colocalized with KRT14, particularly around the periphery of the nucleus, which supported the notion that RIPK4 and KRT14 interact to perform a cellular function. Kinase-dead K51R, which shows a different cellular pattern from the wild type, did not show any colocalization with KRT14 (Figure  2). This indicates that the disruption of kinase activity of RIPK4 does not affect its interaction with KRT14 but disrupts its cellular colocalization with KRT14.KRT14 forms obligate heterodimers with KRT5 at the basal layer of keratinocytes (Fuchs and Weber, 1994). Therefore, we analyzed the effect of RIPK4 on KRT5/KRT14 heterodimer formation and found that RIPK4 specifically interacts with basal keratins through KRT14 but not with KRT5 (Figure  3A), and RIPK4 has no effect on KRT5/KRT14 association (Figures 3B and 3C). The main function of keratin filaments is to provide structural support and to protect cells from mechanical stress (Fuchs and Nowak, 2008). In addition to their well-documented cytoprotective role, keratin filaments participate in various cellular processes such as migration, cell growth, cell division, and differentiation (Kim et al., 2006; Wöll et al., 2007; Busch et al., 2012; Loschke et al., 2015; Sawant and Leube, 2017). These functions of keratin filaments are regulated by dynamic keratin turnover, which is under the control of posttranslational modifications, among which phosphorylation is the major determinant (Snider and Omary, 2014; Kim et al., 2015; Loschke et al., 2015). According to our results, RIPK4 does not show any effect on building up keratin filaments through KRT5/KRT14 heterodimerization; however, as a serine/threonine kinase RIPK4 may regulate keratin turnover through interacting with and phosphorylating KRT14 and/or KRT14-associated proteins. Supporting this idea, a quantitative phosphoproteomics analysis showed that KRT14 phosphorylation was downregulated in RIPK4-deficient cells (Lee et al., 2017). In conclusion, with this study we demonstrated that RIPK4 interacts with the basal keratin network via KRT14. The role of RIPK4/KRT14 interaction in keratinocyte function merits further investigation, which will provide important insight for the understanding of the molecular basis of keratinocyte differentiation and associated skin diseases.

## Acknowledgments 

We thank Dr. Etienne Meylan for the pCR3 VSV-N-RIPK4 and pCR3 VSV-RIPK4 constructs. This study was supported by the Karadeniz Technical University Research Fund (Grant KTÜ-BAP-6717 and BAP-9749 to the third author). We thank Prof. Dr. Ersan Kalay and İdris Er for their technical support in cloning and sequencing experiments. 

## Supplementary Material

(A, B)Analysis of RIPK4 expression in CRISPR/Cas9-applied HaCaT cell line. CRISPR/Cas9 was applied to generate the RIPK4-depleted cell line. The schematic view of selected gRNA sequence preceding PAM (red letter) and its location (between 50th and 68th nucleotides) on corresponding RIPK4 protein is indicated. RIPK4 domains - kinase domain (between 66th and 849th nucleotides, indicated with blue) and ankyrin domain (between 1311th and 2202nd nucleotides, indicated with green) (A). CRISPR/Cas9-induced RIPK4 knockout clone (RIPK4 KO) was analyzed in terms of protein expression by Western blot using anti-RIPK4 antibody (left side). The band corresponding to RIPK4 protein was quantitated and normalized with β-actin (right side). Error bars represent mean ± SD (n = 3). Statistical significance was determined by Student’s t-test (*P < 0.001).
