# Supplementary figures and images for: Keratin 14 is a novel interaction partner of keratinocyte differentiation regulator: receptor-interacting protein kinase 4
Source: Turk J Biol. 2019 Aug 5;43(4):225–34. doi: 10.3906/biy-1904-37 (PMC6713913; doi:10.3906/biy-1904-37)

A

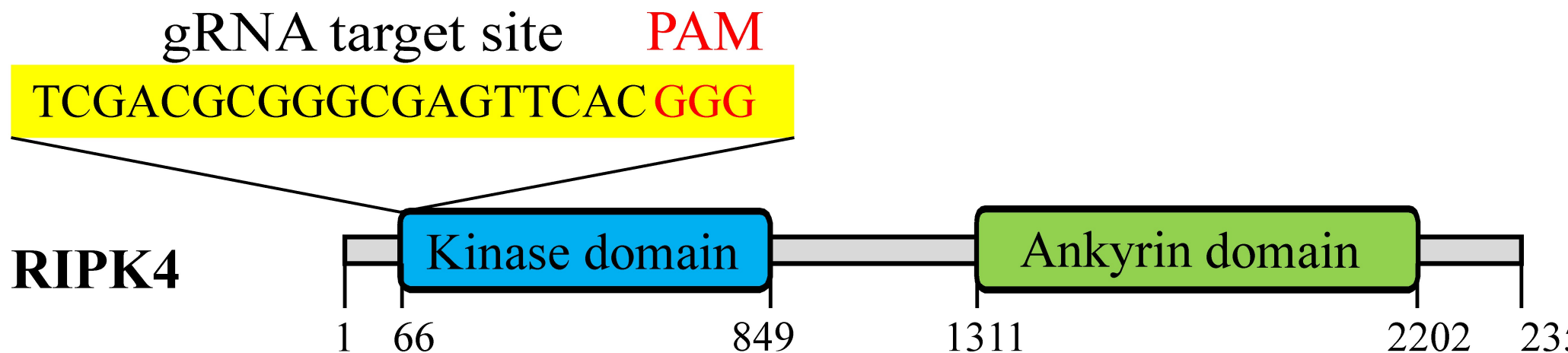

B

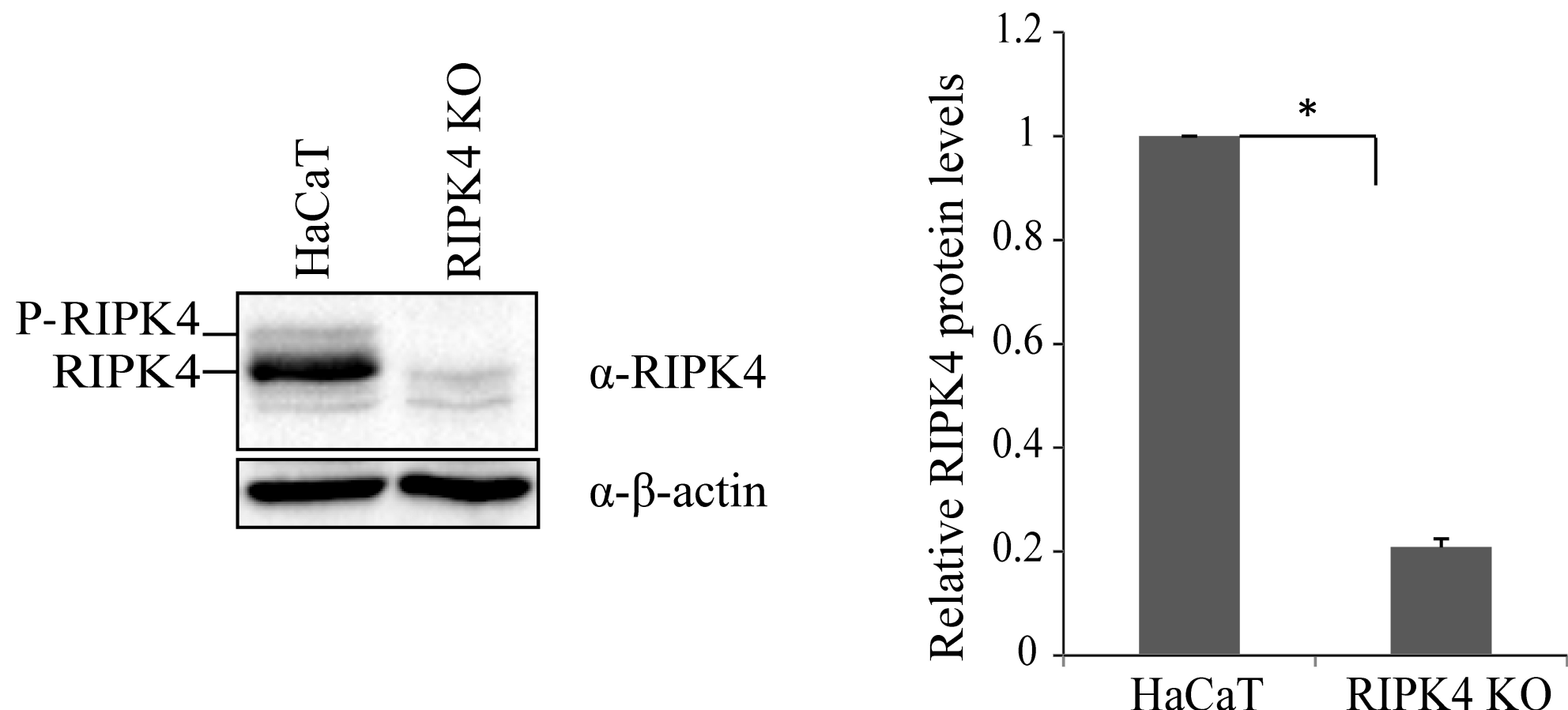

Supplement: (A, B)Analysis of RIPK4 expression in CRISPR/Cas9-applied HaCaT cell line. CRISPR/Cas9 was applied to generate the RIPK4-depleted cell line. The schematic view of selected gRNA sequence preceding PAM (red letter) and its location (between 50th and 68th nucleotides) on corresponding RIPK4 protein is  [file turkjbio-43-225-sup001.pdf]
